# Indirubin Treatment of Lipopolysaccharide-Induced Mastitis in a Mouse Model and Activity in Mouse Mammary Epithelial Cells

**DOI:** 10.1155/2017/3082805

**Published:** 2017-02-01

**Authors:** Jin-lun Lai, Yu-hui Liu, Yong-chong Peng, Pan Ge, Chen-fei He, Chang Liu, Ying-yu Chen, Ai-zhen Guo, Chang-min Hu

**Affiliations:** ^1^The Faculty of Veterinary Medicine, Huazhong Agricultural University, Wuhan 430070, China; ^2^The Faculty of Animal Sciences and Technology, Huazhong Agricultural University, Wuhan 430070, China; ^3^State Key Laboratory of Agricultural Microbiology, Huazhong Agricultural University, Wuhan 430070, China

## Abstract

Indirubin is a Chinese medicine extracted from indigo and known to be effective for treating chronic myelogenous leukemia, neoplasia, and inflammatory disease. This study evaluated the in vivo anti-inflammatory activity of indirubin in a lipopolysaccharide- (LPS-) induced mouse mastitis model. The indirubin mechanism and targets were evaluated in vitro in mouse mammary epithelial cells. In the mouse model, indirubin significantly attenuated the severity of inflammatory lesions, edema, inflammatory hyperemia, milk stasis and local tissue necrosis, and neutrophil infiltration. Indirubin significantly decreased myeloperoxidase activity and downregulated the production of tumor necrosis factor-*α*, interleukin-1*β* (IL-1*β*), and IL-6 caused by LPS. In vitro, indirubin inhibited LPS-stimulated expression of proinflammatory cytokines in a dose-dependent manner. It also downregulated LPS-induced toll-like receptor 4 (TLR4) expression and inhibited phosphorylation of LPS-induced nuclear transcription factor-kappa B (NF-*κ*B) P65 protein and inhibitor of kappa B. In addition to its effect on the NF-*κ*B signaling pathway, indirubin suppressed the mitogen-activated protein kinase (MAPK) signaling by inhibiting phosphorylation of extracellular signal-regulated kinase (ERK), P38, and c-jun NH2-terminal kinase (JNK). Indirubin improved LPS-induced mouse mastitis by suppressing TLR4 and downstream NF-*κ*B and MAPK pathway inflammatory signals and might be a potential treatment of mastitis and other inflammatory diseases.

## 1. Introduction

The occurrence of frequent epidemics of bovine mastitis in dairy herds worldwide causes a heavy cost burden [1, 2]. Mastitis is an inflammatory disease of mammary tissues characterized by a range of physical and chemical changes in milk and pathological changes in udder tissue [3, 4]. There are both noninfectious and infectious forms of mastitis. Infectious mastitis can be caused by microorganisms as diverse as bacteria, yeasts, mycoplasma, and algae, with more than 137 known responsible species [5]. Approximately 80% of bovine mastitis infections are caused by* Escherichia coli *(*E. coli*),* Staphylococcus aureus*,* Streptococcus uberis*,* Streptococcus dysgalactiae*, and* Streptococcus agalactiae *[6, 7].

Acute bovine mastitis with severe clinical symptoms is caused by* E. coli*, most often during early lactation [8]. Lipopolysaccharides (LPS) are a major cell wall component in Gram-negative bacteria. Even transient exposure to LPS is thought to trigger the onset of mastitis in cattle and in mouse models by inducing the production of proinflammatory cytokines [9, 10]. According to the current model, the response to LPS is initialized by specific recognition and binding of agonistic LPS/lipid A on the bacterial cell membrane by LPS binding protein (LBP). LBP is a soluble protein that presents LPS to CD14 and the toll-like receptor 4 (TLR4) *∗* MD-2 complex [11–13]. In activated monocytes and macrophages, MAPK and NF-*κ*B—via transcriptionally active receptor dimers—regulate inflammation by promoting downstream expression of IL-1*β*, IL-6, TNF-*α*, and other cytokines [14].

As there are no effective vaccines against mastitis, disease control primarily depends on antibiotics [15]. However, microbial resistance and the presence of residual antibiotics in milk have hampered mastitis prevention and treatment and threaten food safety [16]. Traditional Chinese medicine is a valuable resource that includes many biologically active components that are both safe and effective. Indirubin, which is extracted from indigo and an active ingredient of Danggui Longhui Wan, is the first traditional Chinese medicine extract shown to be effective for treatment of chronic myelogenous leukemia [17]. Indirubin is derived from several plants, including* Indigofera tinctoria* L.,* Isatis tinctoria* L.,* Cnidii fructus*,* Isatis indigotica*,* Strobilanthes cusia*, and* Polygonum tinctorium* [18]. It acts via suppression of cyclin-dependent kinases (CDKs) and glycogen synthase kinase-3 (GSK-3) activity. Inhibition of the MAPK, NF-*κ*B, p53, B-cell lymphoma 2 (Bcl-2), and wnt/*β*-catenin signaling pathways by indirubin confers antileukemic, antiproliferative, and hepatoprotective properties, antivirus activity, and effectiveness in treating obesity [19–26]. It is also a strong anti-inflammatory agent [27], but the effectiveness of indirubin in treating mastitis is unknown. We evaluated the effectiveness of indirubin against mastitis and investigated the mechanism in LPS-induced MMECs.

## 2. Materials and Methods

### 2.1. Chemicals and Reagents

Indirubin (purity  ≥ 98%) was purchased from Shanghai Yuan Ye Biological Technology Co., Ltd. (Shanghai, China). Dexamethasone Sodium Phosphate Injection (number H37021967) was purchased from Cisen Pharmaceutical Co., Ltd. (Jining, Shandong, China). LPS (*E. coli* 055:B5), human epidermal growth factor, insulin, and transferrin were purchased from Sigma-Aldrich (St. Louis, MO, USA). Fetal bovine serum (FBS) and 0.25% Trypsin-EDTA were purchased from GIBCO (Grand Island, NY, USA). Dulbecco's modified eagle medium (DMEM/F12/1:1) was purchased from Thermo Fisher Biochemical Products Co., Ltd. (Beijing, China). 70 *μ*m nylon mesh filter and 40 *µ*m Falcon cell strainer were purchased from Falcon (Corning, NY, USA). Mouse interleukin-1*β* (IL-1*β*) platinum enzyme-linked immunosorbent assays (ELISA) kits with precoated plates were purchased from eBioscience (San Diego, CA, USA). Mouse IL-6 and TNF-*α* platinum ELISA kits with precoated plates were purchased from BioLegend (San Diego, CA, USA). Mouse myeloperoxidase (MPO) ELISA kits were purchased from MultiSciences (Lianke) Biotech Co., Ltd. (Zhejiang, China). Protein extraction reagent, MTT cell counting kit, HiScript® II RT SuperMix for PCR (R212-01), and AceQ qPCR SYBR Green Master Mix (Q111-02) were purchased from Vazyme Biotech Co., Ltd. (Nanjing, China). TRI Reagent was purchased from Molecular Research Center, Inc. (Cincinnati, Ohio, USA). *β*-Actin (BA2305) was purchased from Wuhan Boster Biological Engineering Co., Ltd. (Wuhan, Hubei, China). Cyclooxygenase-2 (COX-2) (also known as prostaglandin synthase 2) and anti-TLR4 antibody were purchased from Gene Tex, Inc. (San Antonio, TX, USA). BCA Protein Assay Kit, NF-*κ*B pathway sampler kits, and MAPK-family antibody were purchased from Cell Signaling Technology Inc. (Beverly, MA, USA). Bovine serum albumin was purchased from Bio-Sharp (Shanghai, China). Chemiluminescence (ECL) detection kit was come from Advansta (California, USA).

### 2.2. In Vivo Study

#### 2.2.1. Animals

All animal procedures were approved by the Animal Welfare and Research Ethics Committee of Huazhong Agricultural University. Kunming mice (21 males and 42 females) were purchased at 6–8 weeks of age from the Center for Animal Experiment and ABSL-3 Laboratory of WHU (Hubei, China). The mice were maintained for a week to adapt to their new environment, and then one male and two female mice were caged together for a week with free access to water and food. After pregnancy, the female mice were housed separately from the males. The study procedures were carried out 5–7 days postpartum.

#### 2.2.2. Experimental Design

Mice were separated from their offspring 1 h before being anesthetized by intraperitoneal injection (i.p.) of pentobarbital sodium salt (0.25 g pentobarbital sodium salt in 50 mL phosphate-buffered saline (PBS), 10 mg/20 g). The mice were placed in a supine position and the skin around the fourth abdominal mammary glands was sterilized with 75% ethanol. An incision was made about 2 mm proximal of both the L4 and R4 mammary gland teats to expose the udder canal. Anesthetized mice were divided into six groups of eight mice each as follows.

*(1) Control Group (Con)*. 50 *μ*L PBS was injected into the udder canal and 0.2 mL/20 g PBS was given by i.p. injection at 1 h and 12 h.

*(2) DMSO Group (DMSO)*. 50 *μ*L PBS was injected into the udder canal and 0.2 mL/20 g 0.1% DMSO was given by i.p. injection at 1 h and 12 h.

*(3) LPS Group (LPS)*. 50 *μ*L 0.2 mg/mL LPS was injected into the udder canal and 0.2 mL/20 g PBS was given by i.p. injection at 1 h and 12 h.

*(4) Indirubin Administration Groups*. 50 *μ*L 0.2 mg/mL LPS was injected into the udder canal and indirubin was given by i.p. injection of 0.2 mL/20 g of 10 : 1 dilution of 25, 50, or 100 *μ*M at 1 h and 12 h.

*(5) In the Dexamethasone Group (LPS + Dex)*. 50 *μ*L 0.2 mg/mL LPS was injected into the udder canal and 0.5 mg/kg dexamethasone (Dex) was given by i.p. injection at 1 h and 12 h as a positive control.

The mice were euthanized 24 h after LPS challenge, and mammary gland tissue was harvested. The excised mammary gland tissue was photographed and then stored at –80°C for future use.

#### 2.2.3. Tissue Homogenates

Mammary gland tissue was weighed and then homogenized in phosphate buffer (w/v: 1/9) on crushed ice using a tissue grinder. After centrifugation at 2000 g for 40 min at 4°C, the lipid layer was discarded, and the remaining supernatant was centrifuged at 2000 g for 20 min at 4°C to completely remove the lipids. The supernatant was stored at –80°C for future use.

#### 2.2.4. Histopathologic Evaluation and Scoring of Mammary Gland Tissue

Mammary glands were harvested 24 h after LPS challenge for histopathological examination, fixed in 4% paraformaldehyde for 48–72 h, dehydrated in a graded alcohol series, and embedded in paraffin. Histopathological sections were cut into 5 mm thickness and then stained with hematoxylin and eosin (H&E). The scoring of histopathologic changes was done as previously described in studies conducted in mouse mastitis models [28, 29], with minor modifications. The maximum score was 11, and the scoring system is shown in Table 1.

#### 2.2.5. Determination of IL-1*β*, IL-6, TNF-*α*, and MPO Levels

IL-1*β*, IL-6, TNF-*α*, and MPO in mammary gland tissue were assayed in tissue homogenates prepared as described above using ELISA following the kit manufacturer's instructions.

### 2.3. In Vitro Study

#### 2.3.1. Cell Culture and Treatment

MMECs were isolated as previously described with slight modifications [30]. In brief, tissue from the fourth and fifth mouse mammary glands was aseptically harvested from the mice after 18 to 20 days of pregnancy. Tissue fragments were minced into pastes and digested with 0.2% (w/v) each of collagenases I and II in 45 mL DMEM/F12, 5 mL FBS, sterilized by filtering through a 0.45 *μ*m filter, and 0.25% (w/v) Trypsin-EDTA mixture for 3 h at 37°C and 110 rpm in an oscillating incubator. Each digest was centrifuged three times at 250 g for 5 min each and filtered through a 70 *μ*m nylon mesh filter. The filtrates were digested with 0.25% (w/v) Trypsin-EDTA and filtered through a 40 *µ*m Falcon cell strainer to remove epithelial fragments. Primary MMECs were cultured in basic serum-free DMEM/F12 (1 : 1) containing 5 ng/mL EGF, 5 *µ*g/mL insulin, 5 *µ*g/mL transferrin, 10% (v/v) FBS, and 1% (w/v) penicillin-streptomycin, at 37°C in a 5% (v/v) CO_2_ humidified atmosphere.

#### 2.3.2. MTT Assay of Cell Viability

The effect of indirubin on primary MMEC viability was assayed by a standard 3-(4,5-dimethyl-2-thiazolyl)-2,5-diphenyl-2-H–tetrazolium bromide (MTT) assay which was conducted as previously described [31] with slight changes. In short, 1 × 10^4^ MMECs were seeded into 96-well plates, and, 24 h later, with or without LPS (1 *μ*g/mL) addition. 1 h later, indirubin, DMSO < 0.1% v/v, was added and incubation was continued for an additional 24 h. DMSO 0.1% v/v was the control. MTT was added (to 5% w/v) to each well and the culture was continued for 4 h. The medium was removed, and the cells were washed three times with PBS. The formazan crystals were dissolved in 150 *µ*L DMSO/well, and the absorbance was read at 570 nm using a microplate reader.

#### 2.3.3. Enzyme-Linked Immunosorbent Assay

MMECs (1 × 10^6^) were seeded into six-well plates and grown until being 80% to 85% confluent; with or without LPS addition, indirubin, DMSO < 0.1% v/v, was added after 1 h of LPS stimulation and incubation continued for an additional 24 h. Cell-free supernatants were collected for assay of proinflammatory cytokines assays by a mouse ELISA kit, following the manufacturer's instructions.

#### 2.3.4. Total RNA Extraction and Quantitative Real-Time Polymerase Chain Reaction (qRT-PCR)

MMECs (1 × 10^6^ cells) were seeded into six-well plates and grown until being 80% to 85% confluent. With or without LPS addition, indirubin, DMSO < 0.1% v/v, was added after 1 h of LPS stimulation and incubation continued for an additional 24 h. The cells were washed twice with ice-cold PBS; 1 mL of TRIzol reagent was added to each well, following the kit manufacturer's instructions, and the cell lysates were collected. Genomic DNA was isolated from the samples by treatment with 4x gDNA wiper mix, and RNA was reverse-transcribed into cDNA using the HiScript II QRT SuperMix for qPCR, with the gDNA wiper. Relative mRNA concentrations were determined by qRT-PCR using the ViiA (TM) 7 system (Applied Biosystems), SYBR Green Master Mix, and the platinum SYBR Green qPCR SuperMix with 6-carboxyl-X-rhodamine II, following the manufacturer's instructions. The primers used are shown in Table 2 [32–35]. The PCR cycling conditions were 2 min at 50°C followed by 2 min at 95°C, 40 cycles of 15 s at 95°C, 30 s at 58°C, and 30 s at 72°C. Each reaction mixture contained 1 *µ*L cDNA, 5 *µ*L of SYBR Green SuperMix, and sense and antisense primers. Each sample was run in triplicate and the results were averaged. Melting curves were constructed to assess PCR accuracy. 2^−ΔΔ*t*^ method was used to measure the expression levels of calibrator genes. *β*-Actin served as an internal control. We calculated ΔCt values as follows: ΔCt = Ct (target gene) − Ct (housekeeping gene); ΔΔCt = ΔCt (treatment) − ΔCt (control). Amplitude variation served as a surrogate measure of gene expression.

#### 2.3.5. Western Blot Analysis

MMECs (1 × 10^6^) were seeded into six-well plates and grown until being 80% to 85% confluent; with or without LPS addition, indirubin, DMSO < 0.1% v/v, was added after 1 h of LPS stimulation and incubation continued for an additional 24 h. The cells were washed twice with ice-cold PBS, and total proteins were extracted by rapid lysis. Protein extraction reagent was added to lysates on ice, followed by centrifugation at 12,000 g for 10 min to collect supernatants. The proteins were quantitated by the BCA protein assay. Equal aliquots of protein (20–30 *µ*g) were loaded onto a 10% (w/v) sodium dodecyl sulfate-polyacrylamide gel, electrophoresed, and transferred to polyvinylidene difluoride membranes. The membranes were blocked with 5% (w/v) bovine serum albumin with Tris-buffered saline containing 0.05% (v/v) Tween-20 (TBST) at room temperature for 3 h and then washed three times with TBST for 10 min each time. Primary antibodies were diluted in TBST and incubated with the membranes overnight at 4°C with shaking. Membranes were then washed with TBST followed by incubation with Horseradish Peroxidase- (HRP-) conjugated secondary antibody at room temperature for 1 h with shaking. Membranes were developed using an enhanced chemiluminescence (ECL) detection kit and visualized using a chemiluminescence detection system (Chemi Doc, Bio-Rad, USA). Band densities were calculated using ImageJ software (Bio-Techniques, New York, USA).

### 2.4. Statistical Analysis

Data were expressed as means ± SEM and were compared by one-way analysis of variance and Tukey's multiple comparison test, with *p* values < 0.05 considered as statistically significant.

## 3. Results

### 3.1. Macroscopic Pathology and Histological Analysis of Mammary Gland Tissue

**Figure 1:**
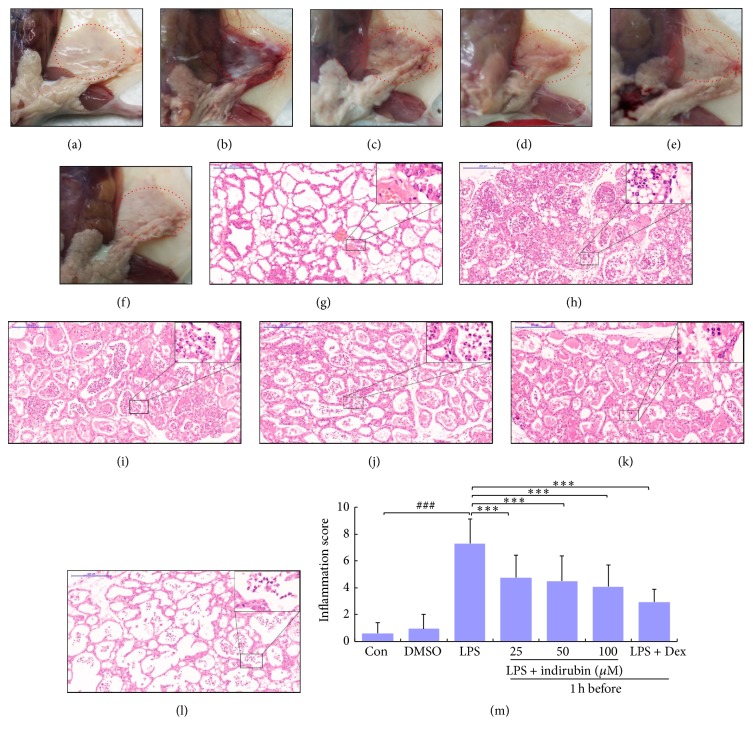
Effect of indirubin on the macroscopic pathology and histological changes in LPS-induced mouse mastitis. Representative macroscopic (a–f) and histological (g–l) changes in mammary glands from the (a, g) control, (b, h) LPS, (c, i) LPS + indirubin (25 *μ*M), (d, j) LPS + indirubin (50 *μ*M), (e, k) LPS + indirubin (100 *μ*M), and (f, l) Dex groups (histological changes observed at 100x magnification; the insets are 400x magnification). (m) Major mammary score (^###^*p* < 0.001 versus control group; ^*∗∗∗*^*p* < 0.001 versus LPS group).

Macroscopic pathology and histological analysis are the most direct methods to evaluate tissue injury and the effect of indirubin treatment. Pathological changes and inflammatory cells were rarely seen in the control (Figures 1(a) and 1(g)) or DMSO groups (data not shown). However, in the LPS group, mammary gland tissue had evident edema, inflammatory hyperemia, milk stasis, and local tissue necrosis (Figure 1(b)). In tissue from the LPS group, mammary alveoli were hyperemic and thicker than in other controls, and neutrophil infiltration was seen in the alveolar lumen (Figure 1(h)). Treatment with indirubin and Dex significantly ameliorated LPS-induced macroscopic changes in a dose-dependent manner (Figures 1(c)–1(e)). Fewer neutrophils and macrophages were seen in the alveolar lumen, the mammary alveoli were thinner, and mammary hyperemia and edema were attenuated of histological, also in a dose-dependent manner (Figures 1(i)–1(k)). Tissue in the LPS group had the highest histological score compared to the control group (*p* < 0.001), and other groups' score was lower than LPS group, especially at a dose of 100 *μ*M (Figure 1(m)).

### 3.2. MPO Activity of Mammary Glands

**Figure 2:**
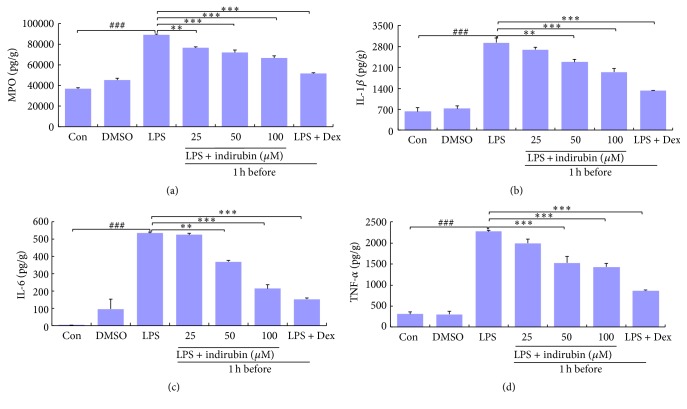
Effect of indirubin on MPO (a), IL-1*β* (b), IL-6 (c), and TNF-*α* (d) in the mammary gland in LPS-stimulated mastitis. Tissue homogenates were used to evaluated MPO (a), IL-1*β* (b), IL-6 (c), and TNF-*α* (d) with ELISA. The values are presented as the means ± SEM of three independent experiments. ^###^*p* < 0.001 versus control group; ^*∗∗*^*p* < 0.01 and ^*∗∗∗*^*p* < 0.001 versus LPS group.

MPO activity was determined to assess neutrophil accumulation within the mammary gland tissue, and is directly proportional to the number of polymorphonuclears within the tissue. As shown in Figure 2(a), MPO was significantly increased (*p* < 0.001) by LPS treatment compared with the control group. Treatment with 25 *μ*M indirubin significantly reduced MPO activity (*p* < 0.01) compared with the LPS group. Interestingly, as the indirubin dose increased, the decrease in MPO activity accelerated. MPO activity was significantly lower in the Dex-treated group than that in the LPS and other treated groups.

### 3.3. Assay of Inflammatory Cytokines in Homogenate Mammary Gland Homogenates

The expression of inflammation cytokines IL-1*β*, IL-6, and TNF-*α* in mammary gland tissue homogenates was measured by ELISA. Compared with the control group, LPS challenge caused a significant increase of all three proinflammatory mediators (*p* < 0.001). Indirubin inhibited the expression of IL-1*β*, IL-6, and TNF-*α* in LPS-induced mouse mastitis in a dose-dependent manner. Expression of all three cytokines was significantly lower than that in the LPS group (*p* < 0.001) but higher than that in Dex-treated mice, in which cytokine expression was also significantly lower than that in LPS group (*p* < 0.001) (Figures 2(b) and 2(c)).

### 3.4. Effect of Indirubin on Cell Viability

**Figure 3:**
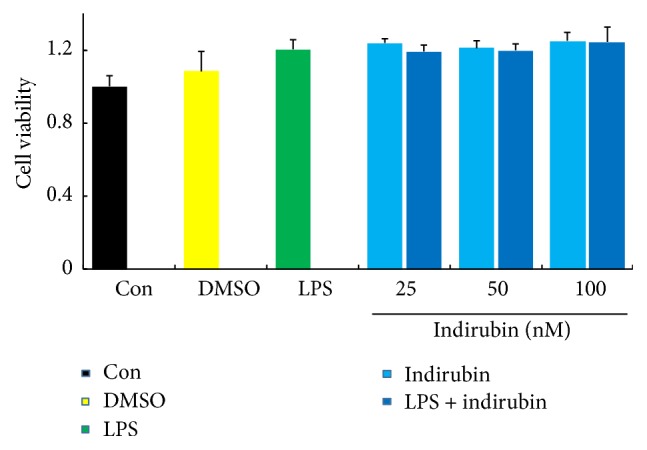
Effects of indirubin on the cell viability in MMECs. The values are presented as the means ± SEM of three independent experiments.

The cytotoxicity of indirubin was determined by MTT assay in the presence or absence of LPS, which also determined the effective concentration used in the experimental procedures. As shown in Figure 3, viability with 0.01% DMSO and with 1 *μ*g/mL LPS, with 25, 50, or 100 nM indirubin, was not significantly different from controls. We also confirmed that MMEC viability with indirubin alone or indirubin plus LPS did not differ. Thus, at concentrations of 0–100 nM indirubin had no observed MMEC cytotoxicity. Consequently, those doses were used in the experimental procedures.

### 3.5. Assay of Inflammatory Cytokines in MMEC

**Figure 4:**
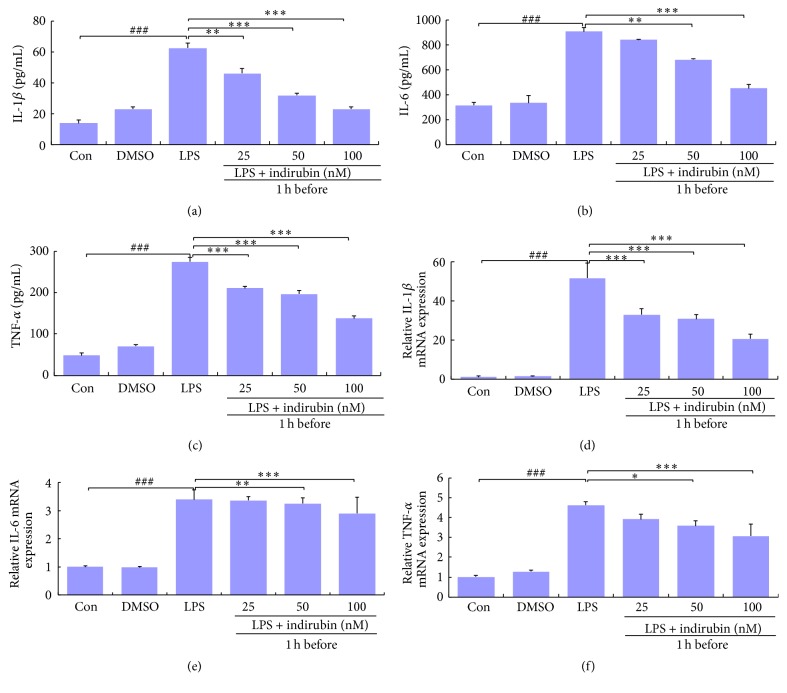
Effects of indirubin on secretion of IL-1*β*, IL-6, and TNF-*α* by LPS-stimulated MMECs. The expressions for IL-1*β* (a, d), IL-6 (b, e), and TNF-*α* (c, f) were measured by ELISA (a–c) and qRT-PCR (d–f). Data are presented as means ± SEM (*n* = 3). ^###^*p* < 0.001 versus control group; ^*∗*^*p* < 0.05, ^*∗∗*^*p* < 0.01, and ^*∗∗∗*^*p* < 0.001 versus LPS group.

ELISA and qRT-PCR were used to determine the effect of indirubin on IL-1*β*, IL-6, and TNF-*α* expression in LPS-induced MMECs. The expression of all three proinflammatory cytokines in LPS-induced MMECs was significantly higher than expression in the control group (*p* < 0.001). Expression in the DMSO and control groups did not differ. Indirubin significantly inhibited IL-1*β*, IL-6, and TNF-*α* expression in MMEC compared with the positive group, and the difference increased significantly with the indirubin dose (Figures 4(a)–4(f)).

### 3.6. Effect of Indirubin on TLR4 Expression

**Figure 5:**
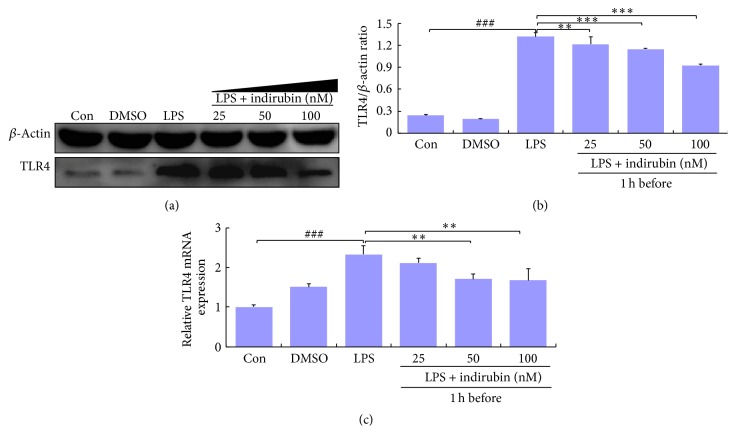
Effects of indirubin on TLR4 expression in LPS-induced MMECs. (a) Cells proteins were analyzed by western blotting. (b) Densitometric analysis of the effects of different concentrations of indirubin on TLR4 expression. (c) mRNA were analyzed by qRT-PCR. Data are presented as means ± SEM (*n* = 3). ^###^*p* < 0.001 versus control group; ^*∗∗*^*p* < 0.01 and ^*∗∗∗*^*p* < 0.001 versus LPS group.

LPS activates the TLR4-mediated NF-*κ*B signaling pathway to trigger the downstream events that regulate cytokine production and the expression of many inflammatory genes. We used qRT-PCR and western blotting to determine whether indirubin inhibited TLR4 expression. Indirubin inhibited TLR4 at 25, 50, and 100 nM, and as the dose increased, TLR4 expression was sharply reduced. The expression of TLR4 mRNA and protein was significantly higher in the LPS group (*p* < 0.001) than in the control group (Figures 5(a)–5(c)).

### 3.7. Indirubin Suppressed the LPS-Induced NF-*κ*B Signaling Pathway

**Figure 6:**
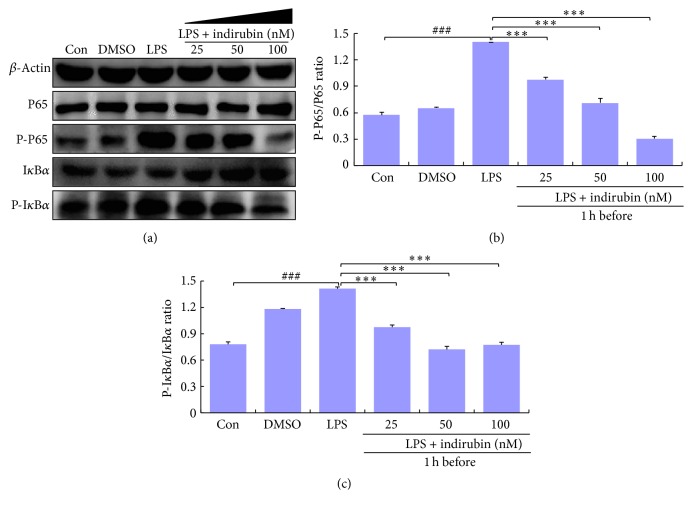
Effects of indirubin on phosphorylation of I*κ*B*α* and NF-*κ*B P65 in LPS-induced MMECs. (a) Cells proteins I*κ*B*α* and NF-*κ*B P65 were analyzed by western blotting. (b) Densitometric analysis of NF-*κ*B P-P65/P65. (c) Densitometric analysis of p-I*κ*B*α*/I*κ*B*α*. The values are presented as means ± SEM (*n* = 3). ^###^*p* < 0.001 versus control group; ^*∗∗∗*^*p* < 0.001 versus LPS group.

Because of its involvement in promoting inflammation, the NF-*κ*B–associated signaling pathway was assessed by western blotting. LPS stimulation significantly increased inhibitor of kappa B (I*κ*B*α*) and P65 phosphorylation compared with the control groups (*p* < 0.001). Indirubin challenge significantly suppressed NF-*κ*B activity in LPS-stimulated MMEC by inhibiting I*κ*B*α* and P65 phosphorylation in a dose-dependent manner (Figures 6(a)–6(c)).

### 3.8. Indirubin Suppressed LPS-Induced Activation of MAPK Pathways

**Figure 7:**
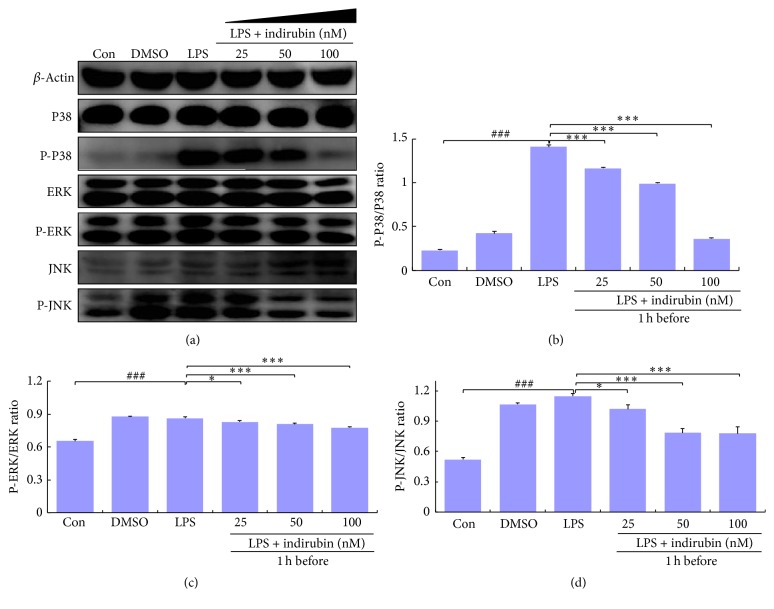
Effects of indirubin on MAPKs expression in LPS-induced MMECs. (a) Cells proteins MAPKs were analyzed by western blotting. (b) Densitometry analysis results of P-P38/P38 expression. (c) Densitometry analysis results of P-ERK/ERK expression. (d) Densitometry analysis results of P-JNK/JNK expression. The cellular proteins were analyzed by western blot and *β*-actin served as internal control. The values are presented as means ± SEM (*n* = 3). ^###^*p* < 0.001 versus control group; ^*∗*^*p* < 0.05 and ^*∗∗∗*^*p* < 0.001 versus LPS group.

The MAPK pathway also mediates proinflammatory gene expression. We assessed indirubin inhibition of inflammation responses via the MAPK pathway in western blots of JNK, ERK, and P38 expression. We found that expression of phosphorylated JNK, ERK, and P38 was significantly increased in LPS-stimulated MMEC compared with the control group (*p* < 0.001). As the indirubin concentration increased, MMEC expression of phosphorylated JNK, ERK, and P38 decreased in a dose-dependent manner, with significant differences from expression in the LPS group (Figures 7(a)–7(d)).

## 4. Discussion

Acute bovine mastitis is characterized by damage to mammary alveoli, edema, inflammatory cell infiltration, and interstitial hemorrhage [36, 37]. LPS induced mouse mammary gland hyperemia, edema, milk stasis, and local tissue necrosis. We observed thickening of the mammary alveolus walls compared with controls and large numbers of inflammatory cells, such as neutrophils and macrophages, within the thickened mammary alveolus. Treatment with indirubin effectively attenuated inflammatory symptoms (Figure 1). MPO activity is a biomarker of tissue infiltration by neutrophils and is directly correlated with the number of infiltrating cells early in the inflammatory process [38]. As shown in Figure 2(a), indirubin treatment significantly reduced MPO activity, which was associated with reduction of neutrophil and macrophage infiltration of the mammary tissue. This confirmed that indirubin had a beneficial effect on the development of the mastitis in the mouse model. Our hypothesis that indirubin inhibited proinflammatory cytokines during LPS-induced acute inflammation in mice mammary tissue led to assay of IL-6, IL-1*β*, and TNF-*α* expression.

Complex, multiple host-pathogen interactions result in accumulation of activated macrophages and large polymorphonuclear neutrophils within the mammary gland to defend against infection. Macrophage activation leads to elimination of the infection and triggers the release of proinflammatory cytokines [39, 40]. The synthesis and release of interferon, TNF, and ILs initiates inflammatory responses and directs neutrophil migration to the site of infection. IL-1*β*, IL-6, and TNF-*α* are proinflammatory cytokines released by activated macrophages, are involved in the upregulation of inflammatory reactions, and are important contributors to the inflammatory response to infection [41–43]. IL-1*β* is released primarily by macrophages, endothelial cells, and monocytes during the inflammatory response at both the local and systemic levels [44, 45]. In the acute-phase of inflammatory reactions, IL-6 has the most important role of the three proinflammatory cytokines. TNF-*α* is an endogenous mediator, with production related to both neuropathic hyperalgesia and inflammation [39, 46]. In our LPS-induced mastitis model, activated macrophages migrated from the mammary interstitium to the alveolar space and generated IL-1*β*, IL-6, and TNF-*α* in the acute stage of inflammation (Figures 2(b)–2(c)).

The MTT assay confirmed that indirubin was not toxic to MMECs up to a concentration of 100 nM (Figure 3). DMSO, at the concentration used in the MTT assay (<0.1% v/v), and LPS at 1 *μ*g/mL were also minimally cytotoxic to MMECs. Those concentrations were thus used in the experimental procedures. ELISA and qRT-PCR confirmed that indirubin suppressed the production of three proinflammatory cytokines. IL-1*β*, IL-6, and TNF-*α* expression significantly increased after LPS stimulation both in MMEC and in the mouse mastitis model. Indirubin treatment significantly inhibited the effect of LPS on both mRNA and protein expression in a dose-dependent manner. LPS activation of macrophages in the mammary alveolar space was also suppressed by indirubin. Collectively, the results showed that indirubin had an anti-inflammatory effect on both LPS-induced MMEC and mouse mastitis. The LPS-induced MMEC inflammation model was used to elucidate the anti-inflammatory mechanism.

As an initial innate defense against infection, TLRs recognize many pathogens and their pathogen-associated molecular patterns and initiate the innate immune response [47, 48]. TLR-pathogen interactions trigger the production of proinflammatory cytokines as well as the functional maturation of antigen-presenting cells of the innate immune system [49, 50]. LPS-induced TLR4 signaling via Myd88-dependent and Myd88-independent pathways activates both the NF-*κ*B and MAPK pathways. This leads to transcription, the induced expression of inducible nitric oxide synthases, COX-2, key inflammatory cytokines such as TNF-*α* and IL-6, and chemokine genes that mount immune and inflammatory responses [51–53]. Western blots and qRT-PCR confirmed that LPS treatment significantly increased expression of both TLR4 protein and mRNA compared with the control group (*p* < 0.001) and that indirubin inhibited LPS-induced TLR4 expression in a dose-dependent manner, especially at 100 nM (Figures 5(a)–5(c)). The results showed that indirubin significantly inhibited LPS-induced TLR4 expression, indicating that the anti-inflammatory effect of indirubin was associated with inhibition of TLR4 activation.

NF-*κ*B has a key role in inflammation and is activated by the TLR4 signal pathway. When not activated, NF-*κ*B exists as homo- or hetero-dimers with P50 and P65 proteins and is bound to I*κ*B*α*. However, when stimulated by stress, oxidized low-density lipoprotein, free radicals, ultraviolet light, or viral and bacterial antigens, I*κ*B*α* kinase phosphorylates NF-*κ*B P65 and ubiquitin-mediated degradation of I*κ*B*α* via the proteasome pathway [54], which promotes translocation of activated NF-*κ*B to the nucleus. In the nucleus, NF-*κ*B promotes the transcription of numerous genes involved in innate and adaptive immune regulation, cell adhesion, inflammatory responses, antiapoptotic mechanisms, and release of proinflammatory cytokines [55, 56]. To clarify the molecular mechanism of IL-*β*, IL-6, and TNF-*α* expression, we measured the impact of indirubin on NF-*κ*B activation and I*κ*B*α* degradation. We found that LPS strongly stimulated phosphorylation of I*κ*B*α* and P65, whereas indirubin suppressed NF-*κ*B activation and degradation of its inhibitor, I*κ*B*α*, in a dose-dependent manner (Figures 6(a)–6(c)). Previous studies demonstrated that magnolol, oxymatrine, selenium, and indirubin-3-monoxime were all able to attenuate inflammatory responses by inhibiting NF-*κ*B activation in LPS-induced mouse mastitis [57–60]. The protective effect of indirubin was similar to that of magnolol, oxymatrine, and taraxasterol but was superior to that of indirubin-3-monoxime.

**Figure 8:**
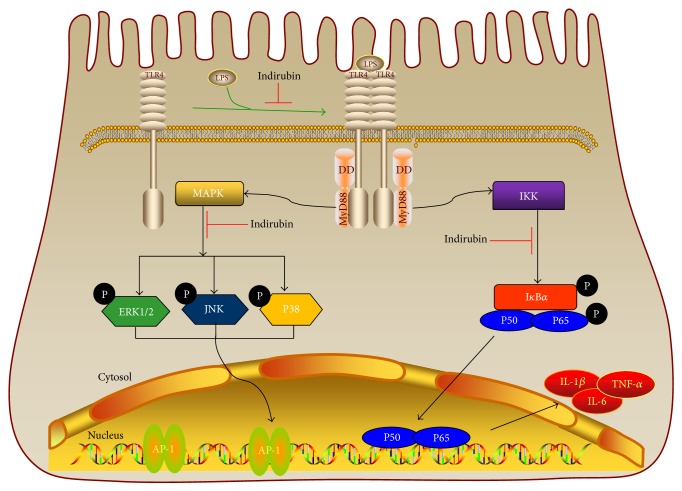
Indirubin treatment of lipopolysaccharide induced mastitis in a mouse model and activity in mouse mammary epithelial cells via bated TLR4 and its two main Myd88-dependent pathways, NF-*κ*B and MAPK.

The TLR4-dependent activation of monocytes/macrophages in response to LPS activates MAPK cascades in addition to NF-*κ*B [61]. The MAPK signaling pathway primarily comprises three ERKs, JNK, and P38 kinase that are present in all eukaryotic cells [62]. Activation of MAPK is followed by activation of transcription factors in the cytoplasm or nucleus, triggering expression of target genes associated with the expression of proinflammatory mediators [61]. As the Myd88-dependent pathway may also have contributed to expression of the proinflammatory cytokines, we determined the levels of ERK, JNK, and P38 expression by western blotting. The results confirmed that indirubin inhibited phosphorylation of ERK, JNK, and P38, in MMEC that had been pretreated with LPS. It is interesting that indirubin suppressed LPS-induced inflammation via its effect on NF-*κ*B in a dose-dependent manner (Figures 7(a)–7(d)). It is thus clear that indirubin treatment of lipopolysaccharide induced mastitis in a mouse model and activity in mouse mammary epithelial cells via bated TLR4 and its two main Myd88-dependent pathways, NF-*κ*B and MAPK (Figure 8).

## 5. Conclusion

Indirubin had a therapeutic effect in LPS-induced mouse mastitis model manifested by attenuation of mammary gland clinical pathology and histopathological changes. It significantly decreased MPO activity, downregulated the expression of IL-1*β*, IL-6, and TNF-*α*, and suppressed TLR4 and downstream events in the NF-*κ*B and MAPKs inflammatory signal pathways. Indirubin has potential as a treatment of mastitis and other inflammatory diseases.

## Figures and Tables

**Table 1:** Histopathologic scoring criteria.

Feature	Description	Score
Hyperemia/edema	Normal	0
	Mild	1
	Moderate	2
	Severe	3

Milk stasis/acinar necrosis	Normal	0
	Mild	1
	Moderate	2
	Severe	3

Infiltration with neutrophil	0-1: acinar or mammary gland neutrophil	0
	2–5: acinar or mammary gland neutrophil	1
	6–10: acinar or mammary gland neutrophil	2
	11–15: acinar or mammary gland neutrophil	3
	16–20: acinar or mammary gland neutrophil	4
	>20: acinar or mammary gland neutrophil	5

**Table 2:** Sequence of primers used in current investigation in qRT-PCR.

Gene	Primers sequence (5′ → 3′)	
*β*-Actin	F: TGCTGTCCCTGTATGCCTCT	R: GGTCTTTACGGATGTCAACG
TNF-*α*	F: CGATGAGGTCAATCTGCCCA	R: CCAGGTCACTGTCCCAGC
IL-1*β*	F: TGAAATGCCACCTTTTGACAG	R: CCACAGCCACAATGAGTGATAC
IL-6	F: TGCCTTCTTGGGACTGAT	R: CTGGCTTTGTCTTTCTTGTT
TLR4	F: TAGCCATTGCTGCCAACATCAT	R: AAGATACACCAACGGCTCTGAA

F: forward. R: reverse.
